# Neglect/abandonment of older women during the COVID-19 pandemic in Brazil: a cross-sectional study

**DOI:** 10.1590/0034-7167-2025-0236

**Published:** 2026-07-24

**Authors:** Carla Maria Kortz Toledo Rodrigues, Dinair Ferreira Machado, Raissa Janine de Almeida, Hugo Fernandes, Mariana Alice Oliveira Ignacio, Ana Paula Pinho Carvalheira

**Affiliations:** I Universidade Estadual Paulista “Júlio de Mesquita Filho”. Botucatu, São Paulo, Brazil; II Universidade Federal de São Paulo. São Paulo, São Paulo, Brazil

**Keywords:** Violence against Women, Elder Abuse, Mandatory Reporting, COVID-19 Pandemic, Brazil., Violencia contra la Mujer, Abuso de Ancianos, Notificación Obligatoria, Pandemia de la COVID-19, Brasil.

## Abstract

**Objectives:**

to analyze factors associated with neglect/abandonment of older women during the COVID-19 pandemic in Brazil.

**Methods:**

a cross-sectional and analytical study of data from the Notifiable Diseases Information System of 2020-2021. Multivariate Poisson regression models were performed using the Statistical Package for the Social Sciences.

**Results:**

5,875 cases of neglect/abandonment were reported. The majority of victims were brown, widowed, without disabilities, with low levels of education, and heterosexual. Factors associated with increased neglect/abandonment were age, having a disability, male perpetrator or both sexes and child or caregiver of victims, homophobia/lesbophobia/biphobia/transphobia, generational conflict and disability, and occurring in an industrial setting. Conversely, factors associated with a decrease in neglect/abandonment were married women/consensual unions and alcohol use by perpetrators.

**Conclusions:**

reporting interpersonal violence during the COVID-19 pandemic allowed for the identification of vulnerabilities and provided support for prevention and early intervention.

## RESUMEN

**Objetivos:**

analizar los factores asociados con la negligencia/abandono de mujeres mayores durante la pandemia de COVID-19 en Brasil.

**Métodos:**

estudio transversal y analítico de datos del Sistema de Información de Enfermedades de Declaración Obligatoria de 2020-2021. Se realizaron modelos multivariantes de regresión de Poisson utilizando el *Statistical Package for the Social Sciences*.

**Resultados:**

se reportaron 5.875 casos de negligencia/abandono. La mayoría de las víctimas eran mestizas, viudas, sin discapacidad, con bajo nivel educativo y heterosexuales. Los factores asociados con un mayor abandono o negligencia fueron la edad, tener una discapacidad, que el agresor fuera hombre o de ambos sexos y el niño o el cuidador de la víctima, la homofobia/lesbofobia/bifobia/transfobia, el conflicto generacional y la discapacidad, y que ocurriera en un entorno industrial. Por el contrario, los factores asociados con una disminución de la negligencia y el abandono fueron las mujeres casadas/uniones consensuales y el consumo de alcohol por parte del agresor.

**Conclusiones:**

la denuncia de la violencia interpersonal durante la pandemia de COVID-19 permitió identificar vulnerabilidades y brindó apoyo para la prevención y la intervención temprana.

## INTRODUCTION

In 2020, at the height of the COVID-19 pandemic, the United Nations General Assembly declared the start of the Decade of Healthy Ageing 2020-2030. This period offers an opportunity to bring together governments, civil society, professionals, academic institutions, the media, and the private sector around ten years of action in favor of an age-equitable society in all nations around the world, with the aim of improving the lives of older people, their families, and the communities in which they live^(1)^.

The population is aging worldwide more rapidly than in the past, and this demographic transition will affect almost every aspect of society. Globally, there are already more than one billion people aged 60 or older, and most of them live in lowand middle-income countries. Many of these people lack even access to the basic resources necessary for a full and dignified life, facing numerous obstacles that prevent them from fully participating in society^(1)^. Brazil has more than 31 million older adults, representing 16% of the population. According to projections from the Brazilian Institute of Geography and Statistics, the percentage of older adults is expected to double in the coming decades^(2)^. Women are the majority among older adults. Many experience old age in conditions of loneliness and face challenges such as lack of financial resources, making it difficult to meet their basic needs, making them more susceptible to various forms of violence and increasing the vulnerability of this group^(3)^.

In this regard, older women face double vulnerability with regard to violence, stemming from both age and gender. Oppression and domination related to gender inequality, present in all phases of life, intensify in old age^(4-6)^.

According to a meta-analysis published by The Lancet Global Health, approximately one in six older adults worldwide is a victim of some type of violence, corresponding to a prevalence of 15.7%, with 11.6% being psychological violence, 6.8% financial abuse, 2.6% physical violence, 0.9% sexual violence, and 4.2% neglect/abandonment^(7)^.

Elder abuse, manifested primarily through neglect and abandonment, reveals the structural flaws of a society that has historically marginalized both older adults and women, forming a highly vulnerable group susceptible to external factors, such as the COVID-19 pandemic, which intensified rates of violence against women and older adults^(8-10)^.

With the advent of COVID-19, several actions had to be taken due to the high rate of contagion of the virus. To reduce the speed of transmission, a series of interventions were implemented, such as social distancing and isolation^(11)^. In this context, lockdowns have been shown to exacerbate violence against women and older adults, leading to an exponential increase worldwide^(12,13)^.

Regarding violence against women, countries such as Argentina, Canada, France, Germany, the United Kingdom, and the United States reported an increase in cases and demand for emergency shelters and support networks. In the United Kingdom, Australia, and France, reports of violence rose by 65%^(14)^. In India, reports doubled in the first week of social distancing^(15)^. Latin American countries, such as Bolivia, Colombia, Argentina, and Brazil, have also recorded an increase in domestic violence^(16)^. In Brazil, the Call 180 hotline recorded a 35.9% increase in the number of calls received in April 2020, totaling 9,842 calls compared to April 2019^(17)^.

During the pandemic, there was a tenfold increase in abuse and neglect against older adults, especially those with mobility issues and difficulties accessing information^(6)^. However, despite the alarming increase, the topic is still understudied, with a lack of research and discussions that specifically address the issue^(4,8,18)^.

It is undeniable that the social isolation imposed by the pandemic accentuated the structural weaknesses of society, leading to a significant increase in cases of neglect and abandonment, particularly among older women. However, such phenomena still require further scientific investigation, and the available literature presents significant gaps regarding elder abuse during the pandemic period, especially concerning older women, who face additional challenges due to the intersection of age and gender^(3,13,19)^.

Given the above, the following question arose for the authors: what aspects are associated with neglect/abandonment of older women during the COVID-19 pandemic?

## OBJECTIVES

To analyze the factors associated with neglect/abandonment of older women during the COVID-19 pandemic in Brazil.

## METHODS

### Ethical aspects

The preservation of ethical aspects was ensured in accordance with Resolution of the Brazilian National Health Council, dated April 7, 2016, sole paragraph, which states that research that uses publicly available information will not be registered or assessed by the Research Ethics Committee/National Research Ethics Commission (In Portuguese, *Comitê de Ética em Pesquisa/Comissão Nacional de Ética em Pesquisa* - CEP/CONEP) system, in item II, under the terms of Law 12,527 of November 18, 2011.

It is important to note that the database used is publicly accessible and does not contain the names of participants or any other possibility of individually identifying women, thus guaranteeing anonymity. Therefore, it was not necessary to refer the case to an Ethics Committee for review.

### Study design, period and location

This is a cross-sectional^(20)^ and analytical study, guided by the STrengthening the Reporting of OBservational studies in Epidemiology^(21)^, developed from secondary data from the Notifiable Diseases Information System (In Portuguese, *Sistema de Informação de Agravos de Notificação* - SINAN), obtained from the of Brazilian Health System Department of Informatics (In Portuguese, *Departamento de Informática do Sistema Único de Saúde* - DATASUS) and accessed through TabNet on March 4, 2024.

### Population, inclusion and exclusion criteria

All cases of neglect/abandonment against older women that occurred in Brazil and were registered in SINAN between 2020 and 2021 were included. Notifications in which the victim’s gender was “ignored” and suspected or confirmed cases of self-inflicted violence were excluded.

### Study protocol

Data were collected using information from the notification forms for suspected or confirmed cases of interpersonal violence in the SINAN system, made available by the Brazilian National Health Surveillance Department through TabNet, a program developed by DATASUS, and are accessible online without access restrictions. At the time of the search, the system had data available up to the year 2022.

Chart 1 presents and analyzes variables related to sociodemographic characteristics of older women, perpetrators, and neglect/abandonment.

**Chart 1:** Study variables according to type and expression, Brazil, 2020-2021

Variable	Type	Expression
**Sociodemographic characteristics of older women**		
Race/color	Categorical	White; black; yellow; brown; indigenous; blank.
Education (complete years)	Categorical	Illiterate; 1^st^ to 4^th^ grade incomplete of elementary school; 4^th^ grade incomplete of elementary school; 5^th^ to 8^th^ grade incomplete of elementary school; complete elementary school; incomplete high school; complete high school; incomplete higher education; complete higher education; ignored.
Marital status	Categorical	Single; married/common-law marriage; widow; separated; blank.
Do you have any type of disability/disorder?	Dichotomous	Yes; no.
Sexual orientation	Categorical	Heterosexual; homosexual; bisexual; blank.
**Characteristics of perpetrators and of negligence/abandonment**		
Perpetrator’s sex	Categorical	Female; male; both sexes.
Suspected alcohol use	Dichotomous	Yes; no.
Perpetrator’s life cycle	Categorical	Child (0-9 years); adolescent (10-19 years); young person (20-24 years); adult (25-59 years); older adult (≥60 years).
Location of occurrence	Categorical	Residence; collective housing; school; sports practice venue; bar or similar; public road; trade/services; industry/construction.
Motivation for violence	Categorical	Sexism; homophobia/lesbophobia/biphobia/transphobia; racism; religious intolerance; xenophobia; generational conflict; living on the street; disability; others.
Perpetrator’s relationship with the older woman	Categorical	Father; mother; stepfather; stepmother; partner; ex-partner; boyfriend/girlfriend; ex-boyfriend/girlfriend; child; unknown person; sibling, acquaintance/friend; caregiver; boss/employer; institutional; police officer/law enforcement agent.

### Analysis of results and statistics

Descriptive analyses of sociodemographic variables of older women, perpetrators, and neglect/abandonment were performed. The investigation of factors associated with neglect/abandonment of older women in the pandemic context was carried out by fitting multiple regression models with Poisson response in two stages. In the first stage, a bivariate regression model was fitted, including all explanatory variables in the deterministic component. Variables that showed an association with p<0.05 were considered in the second stage, which consisted of fitting a new multiple linear regression model with Poisson response only with the variables identified in the previous stage. In the final model, the results were expressed as prevalence ratios, with 95% Confidence Intervals and p-values, considering p<0.05 as the level of statistical significance.

For data tabulation and analysis, Microsoft Excel 2007 and the Statistical Package for the Social Sciences were used, respectively.

## RESULTS

During the period outlined, 5,875 cases of older women who suffered neglect/abandonment were reported nationwide. According to Table 1, it was observed that 48.8% (n=2.867) of women identified as brown; 12.8% (n=753) had incomplete elementary school and high school; 28.7% (n=1.684) were widows; 78.4% (n=4.395) had no disability/disorder; and 63.7% (n=3.740) were heterosexual.

**Table 1:** Reports of neglect/abandonment against older women according to sociodemographic characteristics of victims, Brazil, 2020-2021

Sociodemographic characteristics	n	%
**Race/color**		
White	2,212	37.7
Black	431	7.3
Yellow	43	0.7
Brown	2,867	48.8
Indigenous	30	0.5
Blank	292	5.0
**Education**		
Illiterate	314	5.3
1st to 4th grade incomplete of elementary school	753	12.8
4th grade incomplete of elementary school	286	4.9
5th to 8th grade incomplete of elementary school	306	5.2
Complete elementary school	225	3.8
Incomplete high school	74	1.3
Complete high school	119	2.0
Incomplete higher education	9	0.2
Complete higher education	52	0.9
Ignored	3,737	63.6
**Marital status**		
Single	720	12.3
Married/common-law marriage	841	14.3
Widow	1,684	28.7
Separated	271	4.6
Blank	2,659	40.2
Do you have any type of disability/disorder?		
No	4,395	74.8
Yes	1,480	25.2
**Sexual orientation**		
Heterosexual	3,740	63.7
Homosexual	21	0.4
Bisexual	4	0.1
Blank	2,110	35.9

*Source: Notifiable Diseases Information System, 2020 - 2021.*

According to Table 2, 44.1% (n=2,589) of neglect/abandonment cases were perpetrated by both males and females. Regarding perpetrator(s), 62.6% (n=3.676) were adults (25 to 59 years old); 54.9% (n=3.225) were not suspected of alcohol use; 69.3% (n=4.069) had a child relationship; and 5.7% (n=337) had a caregiver relationship with the victim. Considering the location of the incident, 80.5% (n=4.728) occurred in the residence, with 47.6% (n=2.798) due to other reasons, and 9.6% (n=563) due to generational conflict.

**Table 2:** Reports of neglect/abandonment against older women according to perpetrator and violence characteristics, Brazil, 2020 - 2021

Characteristics	n	%
**Perpetrator’s sex**		
Male	1,186	20.2
Female	1,241	21.1
Both sexes	2,589	44.1
Blank	859	14.6
**Perpetrator’s life cycle**		
Child (0 to 9 years)	27	0.5
Adolescent (10 to 19 years)	52	0.9
Young person (20 to 24 years)	144	2.5
Adult (25 to 59 years)	3,676	62.6
Older adult (≥60 years)	606	10.3
Blank	1,370	23.3
**Suspected alcohol use**		
No	3,225	54.9
Yes	466	7.9
Blank	2,184	37.2
**Perpetrator’s relationship with the older woman**		
Father	20	0.3
Mother	51	0.9
Stepfather	1	0.0
Stepmother	1	0.0
Partner	277	4.7
Ex-partner	22	0.4
Boyfriend/girlfriend	4	0.1
Ex-boyfriend/girlfriend	10	0.2
Child	4,069	69.3
Sibling	483	4.8
Acquaintance	126	2.1
Unknown person	29	0.5
Caregiver	337	5.7
Boss	6	0.1
Institutional	77	1.3
Police officer/law enforcement agent	1	0.00
**Location where neglect/abandonment occurred**		
Residence	4,728	80.5
Collective housing	102	1.7
School	0	0.0
Sports practice venue	2	0.0
Bar or similar	1	0.0
Public road	135	2.3
Trade/services	150	2.6
Industries/construction	1	0.0
Blank	756	12.9
**Motivation for neglect/abandonment**		
Sexism	69	1.2
Homophobia/lesbophobia/biphobia/transphobia	21	0.4
Racism	2	0.0
Religious intolerance	3	0.1
Xenophobia	4	0.1
Generational conflict	563	9.6
Living on the street	11	0.2
Disability	89	1.5
Others	2,798	47.6
Blank	2,315	39.4

*Fonte: Sistema de Informação de Agravos de Notificação, 2020 - 2021.*

**Table 3:** Bivariate associations using simple Poisson regression to explain neglect/abandonment against older women during the pandemic, Brazil, 2020-2021

Variables	PR	CI	*p* value
**Age**	1.05	1.05-1.06	**0.000**
**Race/color**			
Indigenous	0.80	0.56-1.15	0.230
Brown	1.53	1.45-1.62	**0.000**
Yellow	1.20	0.89-1.62	0.241
Black	1.02	0.92-1.13	0.742
White	1		
**Education**			
1st to 4th grade incomplete of elementary school	0.91	0.79-1.03	**0.000**
4th grade incomplete of elementary school	0.79	0.68-0.93	**0.000**
5th to 8th grade incomplete of elementary school	0.64	0.55-0.75	**0.000**
Complete elementary school	0.62	0.55-0.73	**0.000**
Incomplete high school	0.42	0.33-0.54	**0.000**
Complete high school	0.29	0.23-0.35	**0.000**
Incomplete higher education	0.20	0.10-0.39	**0.004**
Complete higher education	0.36	0.27-0.48	0.143
Illiterate	1		
**Marital status**			
Separated	0.57	0.50-0.66	**0.000**
Widow	1.33	1.22-1.45	**0.000**
Married/common-law marriage	0.56	0.51-0.62	**0.000**
Single	1		
**Do you have any type of disability/disorder?**			
Yes	1.90	1.79-2.02	**0.000**
No	1		
**Sexual orientation**			
Bisexual	0.80	0.30-2.13	0.655
Homosexual	0.54	0.35-0.82	**0.004**
Heterosexual	1		
**Perpetrator’s sex**			
Both sexes	7.37	6.88-7.90	**0.000**
Male	2.98	2.75-3.23	**0.000**
Female	1		
**Perpetrator’s life cycle**			
Older adult (≥60 years)	0.51	0.34-0.74	**0.001**
Adult (25 to 59 years)	0.91	0.62-1.33	0.632
Young person (20 to 24 years)	0.38	0.25-0.57	**0.000**
Adolescent (10 to 19 years)	0.39	0.24-0.62	**0.000**
Child (0 to 9 years)	1		
**Alcohol use**			
Yes	0.23	0.21-0.25	**0.000**
No	1		
**Perpetrator’s relationship with the older woman**			
Father	0.88	0.57-1.36	0.557
Mother	1.11	0.84-1.46	0.454
Stepfather	0.26	0.04-1.87	0.182
Stepmother	0.26	0.04-1.87	0.182
Sibling	1.27	1.12-1.43	**0.000**
Partner	0.26	0.23-0.29	**0.000**
Ex-partner	0.10	0.06-0.15	**0.000**
Boyfriend/girlfriend	0.09	0.03-0.24	**0.000**
Ex-boyfriend/girlfriend	0.32	0.17-0.60	**0.000**
Child	3.94	3.70-4.19	**0.000**
Friend/acquaintance	0.31	0.26-0.37	**0.000**
Unknown person	0.09	0.07-0.14	**0.000**
Caregiver	2.64	2.37-2.95	**0.000**
Boss/employer	0.73	0.33-1.63	0.446
Institutional	2.07	1.66-2.60	**0.000**
Police officer/law enforcement agent	0.11	0.02-0.81	**0.000**
**Motivation for neglect/abandonment**			
Others	17.71	13.95-22.49	**0.000**
Disability	14.88	10.87-20.38	**0.000**
Living on the street	3.02	1.60-5.71	**0.001**
Generational conflict	6.47	5.04-8.31	**0.000**
Xenophobia	12.26	4.47-33.60	**0.000**
Religious intolerance	6.90	2.17-21.91	**0.001**
Racism	4.60	1.13-18.75	**0.033**
Homophobia/lesbophobia/biphobia/transphobia	14.85	9.11-24.21	**0.000**
Sexism	1		
**Location where neglect/abandonment occurred**			
Industries/construction	1.70	0.24-12.07	0.596
Trade/services	1.81	1.54-2.13	**0.000**
Public road	0.51	0.43-0.60	**0.000**
Bar or similar	0.05	0.01-0.33	**0.002**
Sports practice venue	0.38	0.09-1.51	0.169
School	#		
Collective housing	1.95	1.60-2.37	**0.000**
Residence	1		

*PR - Poisson Regression; CI - Confidence Interval; #Estimate not obtained due to convergence problems of the maximum likelihood estimator, i.e., estimate not obtained for calculation reasons.*

*Source: Notifiable Diseases Information System, 2020 - 2021.*

In the bivariate regression analysis (Table 3), independently, there was a significant association between neglect/abandonment against older women during the pandemic, with its occurrence increasing with the following variables: being an older adult; being brown; being widowed; having some type of disability/disorder; the perpetrator being male or of both sexes; the relationship between the perpetrator and the victim being child, sibling, caregiver, and institutional; the motivation that led to neglect/abandonment being due to others, disabilities, living on the street, generational conflict, xenophobia, religious intolerance, racism, and homophobia/lesbophobia/biphobia/transphobia; and the incident having occurred in trade/services and collective housing.

**Table 4:** Multiple Poisson regression analysis to explain neglect/abandonment against older women during the pandemic, Brazil, 2020-2021

Variables	PR	CI	*p* value
**Age**	1.03	1.02-1.04	**0.000**
**Race/color**			
Indigenous	0.85	0.33-2.12	0.728
Brown	1.15	0.95-1.40	0.137
Yellow	0.56	0.79-4.08	0.574
Black	1.05	0.78-1.43	0.715
White	1		
**Education**			
1st to 4th grade incomplete of elementary school	0.80	0.45-1.45	0.480
4th grade incomplete of elementary school	0.54	0.13-2.25	0.404
5th to 8th grade incomplete of elementary school	0.77	0.51-1.15	0.212
Complete elementary school	0.72	0.42-1.24	0.244
Incomplete high school	0.97	0.66-1.42	0.881
Complete high school	1.02	0.72-1.43	0.902
Incomplete higher education	1.01	0.73-1.40	0.911
Complete higher education	1.06	0.81-1.38	0.658
Illiterate	1		
**Marital status**			
Separated	0.71	0.50-1.01	0.060
Widow	0.78	0.61-1.00	0.054
Married/common-law marriage	0.69	0.52-0.93	**0.015**
Single	1		
**Do you have any type of disability/disorder?**			
Yes	1.75	1.31-2.33	**0.000**
No	1		
**Sexual orientation**			
Bisexual	0.53	0.07-3.88	0.535
Homosexual	1.13	0.41-3.06	0.808
Heterosexual	1		
**Perpetrator’s sex**			
Both sexes	2.02	1.52-2.67	**0.000**
Male	1.51	1.20-1.89	**0.000**
Female	1		
**Perpetrator’s life cycle**			
Older adult (≥60 years)	1.46	0.20-10.69	0.708
Adult (25 to 59 years)	1.48	0.20-10.70	0.697
Young person (20 to 24 years)	0.89	0.11-6.85	0.918
Adolescent (10 to 19 years)	0.94	0.10-8.31	0.961
Child (0 to 9 years)	1		
**Alcohol use**			
Yes	0.39	0.30-0.50	**0.000**
No	1		
**Perpetrator’s relationship with the older woman**			
Sibling	1.28	0.83-1.99	0.255
Partner	1.07	0.70-1.63	0.740
Ex-partner	0.60	0.21-1.67	0.334
Boyfriend/girlfriend	#		
Ex-boyfriend/girlfriend	1.69	0.23-12.38	0.601
Child	2.52	1.92-3.30	**0.000**
Friend/acquaintance	0.66	0.40-1.08	0.099
Unknown person	0.45	0.16-1.25	0.126
Caregiver	1.52	1.05-2.19	**0.025**
Boss/employer	#		
Institutional	1.63	0.34-7.78	0.539
Police officer/law enforcement agent	#		
**Motivation for neglect/abandonment**			
Others	5.51	3.24-9.36	**0.000**
Disability	4.98	2.45-10.10	**0.000**
Living on the street	1.38	0.17-11.02	0.756
Generational conflict	3.78	2.20-6.50	**0.000**
Xenophobia	#		
Religious intolerance	4.30	0.56-33.98	0.160
Racism	5.45	0.70-46.45	0.105
Homophobia/lesbophobia/biphobia/transphobia	5.81	1.65-20.46	**0.006**
Sexism	1		
**Location where neglect/abandonment occurred**			
Industries/construction	8.68	1.18-63.87	**0.034**
Trade/services	1.35	0.72-2.53	0.335
Public road	1.11	0.65-1.91	0.686
Bar or similar	#		
Sports practice venue	#		
School	#		
Collective housing	1.17	0.43-3.18	0.746
Residence	1		

*PR - Poisson Regression; CI - Confidence Interval; #Estimate not obtained due to convergence problems of the maximum likelihood estimator, i.e., estimate not obtained for calculation reasons.*

*Source: Notifiable Diseases Information System, 2020 - 2021.*

On the other hand, also independently, there was a significant association between neglect/abandonment against older women during the pandemic, decreasing its occurrence, and the following variables: being women with education from 1^st^ to 4^th^ grade to incomplete higher education; being an older woman who was separated or married/in a common-law marriage; being homosexual; in relation to the perpetrator’s life cycle, being an older adult (≥60 years), a young person (20 to 24 years) and an adolescent (10 to 19 years); being intoxicated at the time of the incident; the relationship between the perpetrator and the victim being that of a partner, ex-partner, boyfriend/girlfriend, ex-boyfriend/girlfriend, police officer/law enforcement agent, or friend/acquaintance; and the incident having occurred on a public road and in a bar or similar setting.

In the multiple regression analysis (Table 4), the following variables independently maintained a significant association with neglect/abandonment against older women during the pandemic, increasing its occurrence: being an older adult; having some type of disability/disorder; the perpetrator being male or of both sexes; the relationship between the perpetrator and the victim being child or caregiver; the reasons for neglect/abandonment being homophobia/lesbophobia/biphobia/transphobia, generational conflict, disabilities, and others; and the violence occurring in an industrial setting. On the other hand, also independently, the following variables maintained a significant association with neglect/abandonment against older women during the pandemic, decreasing its occurrence: being married or in a consensual union; and the perpetrator being suspected of alcohol use.

## DISCUSSION

Evidence suggests that older women who are dependent on others for activities of daily living or who have dementia are more susceptible to abuse and neglect, as the burden on caregivers, often without adequate training, can lead to neglect or abandonment, whether intentional or not. Older adults with neurocognitive disorders, in particular, are a high-risk population for abuse in the context of caregiving. These chronic health conditions, along with physical and cognitive limitations, increase these women’s vulnerability to violence^(8,9,22-24)^.

During the pandemic, the occurrence of violence committed by male perpetrators increased, as observed in this research. A study in São Paulo indicated that 56.7% of cases of elder abuse in the first year of the pandemic involved male perpetrators, with frustration due to job loss or economic hardship frequently being directed at older women^(9)^. However, women can also be perpetrators of violence, especially when faced with emotional and economic overload in caring for older women. In this context, caregivers without adequate support often neglect their responsibilities, resulting in involuntary abandonment^(25)^.

However, in most cases, caregivers are female family members, generally daughters or daughters-in-law with a close and affectionate bond with older woman. Caregiving work is often continuous and solitary, without the support of services or public assistance policies. This role imposes limitations on caregivers’ personal life, leading to overload, unemployment, and distancing from the social and emotional network, generating conflict within the family and with older woman. Gender inequality clearly permeates these relationships, because at some point, this older woman was the one responsible for family care, and upon transitioning to a dependent care status, she becomes useless to the system and a burden on the family^(26)^.

This pattern reflects the feminization of old age, in which older women live longer but face greater functional dependence and, consequently, a higher incidence of abandonment and neglect. Thus, the pandemic context showed that both sexes can act as perpetrators, although the dynamics and motivations may vary^(24)^.

Another aspect related to violence is prejudice related to sexual orientation and gender identity. Violence against older women who are Lesbian, Gay, Bisexual, Transgender/Transvestite, Queer, Intersex, Asexual, and of other sexual orientations and gender identities is intensified by prejudices such as homophobia and transphobia, which affect their access to care, especially in family and healthcare settings. During the pandemic, these discriminations increased, resulting in greater family rejection and abandonment, as well as social isolation, which exacerbated exclusion from support services. The aging of these women, coupled with age-related stigmas and lack of family and social acceptance, makes them more vulnerable to neglect and abandonment. During the pandemic, social invisibility and the lack of specific protection policies further amplified this situation^(13,27)^.

Considering the location where neglect/abandonment occurred in the current research, and although there are no recent studies that directly identify industries as factors that increase the prevalence of neglect/abandonment in older women, workplace violence, including managerial abandonment, affects the retention of older women in the workforce. The lack of preparedness of companies to deal with an aging workforce and negative stereotypes result in discrimination and early departure, constituting structural violence. During the COVID-19 pandemic, these factors worsened, especially for older women, who faced greater vulnerability to abandonment at work and in their families due to social isolation and economic hardship^(28)^.

Throughout the pandemic, the occurrence of neglect/abandonment was lower among older women who were married or in stable relationships, as the presence of a partner often provides emotional and practical support. Marital status may be a factor in reducing the occurrence of neglect, as couples tend to develop mutual caregiving relationships, which can mitigate the occurrence of abandonment. Older women living with their partners reported less emotional isolation, as their partner was present to offer immediate support. Furthermore, the financial stability of many couples contributed to maintaining access to essential services during the pandemic, reducing the occurrence of neglect^(8)^.

The variable of suspected alcohol use by the perpetrator of violence was also associated with a lower prevalence of neglect/abandonment. This suggests that family structure and contexts surrounding alcohol consumption may influence the neglect/abandonment dynamics. A possible explanation is that, during the pandemic, there were restrictions on the operation of bars and other public establishments. Therefore, alcohol consumption, which previously took place in such spaces, moved to private settings, with the home becoming the preferred location for this practice^(29)^.

### Study limitations

The main limitation of this study includes the possibility of an increase in late notifications of interpersonal violence cases in SINAN, which may not be included in the information collected in this study. Furthermore, a high number of notifications were marked as “ignored” or “blank”. This type of occurrence is considered a failure in completing the mandatory notification form for suspected or confirmed cases of violence, as it significantly affects the accurate characterization of the victims’ profiles and the appropriate adoption of protection and intervention measures^(30)^.

Another relevant finding was that not all the information present in the mandatory notification form was tabulated by the system, such as the outcomes or referrals of the cases. However, this weakness does not compromise the results, as relevant information is presented about the characteristics of neglect and abandonment suffered by older women during the COVID-19 pandemic in Brazil. Finally, limiting data collection to the years 2020 and 2021, as they represent the pandemic period, makes it impossible to analyze changes in the post-pandemic period, highlighting the relevance of further investigations.

### Contributions to nursing, health, or public policy

The importance of mandatory reporting of interpersonal violence during the COVID-19 pandemic is highlighted, as it made it possible to identify older women with greater vulnerability, promoting resources for prevention and early intervention in cases of elder abuse.

Given the high number of variables with “blank” and “ignored” data found, it is suggested that ongoing education is needed throughout the country on the correct completion of the notification form for suspected or confirmed cases of violence.

## CONCLUSIONS

This study enabled the analysis of factors associated with neglect/abandonment of older women during the COVID-19 pandemic in Brazil. The increase in its occurrence was significantly associated with the woman’s age, presence of disability/disorder, perpetrator profile (male or both sexes), and relationship with the victim, especially when it involved a child or caregiver. Motivations such as homophobia/lesbophobia/biphobia/transphobia, generational conflicts, and disability were also associated, as was the occurrence in industrial settings. On the other hand, being married/in a common-law marriage and suspicion of alcohol use by the perpetrator independently decreased the occurrence of this problem.

These findings reinforce the need for intersectoral strategies that consider the specificities of this profile, promoting public policies aimed at protecting and caring for older women in vulnerable contexts.

## Data Availability

The research data are available only upon request.
