# Supplementary material for: Neglect/abandonment of older women during the COVID-19 pandemic in Brazil: a cross-sectional study
Source: Rev Bras Enferm. 2026 Jul 24;79:e20250236. doi: 10.1590/0034-7167-2025-0236 (PMC13403640; doi:10.1590/0034-7167-2025-0236)
Supplement: Translation

# Translation: Negligência/abandono de mulheres idosas durante a Negligencia/abandono de mujeres mayores durante la pandemia de COVID-19 en Brasil: un estudio transversal

**DOI:** 10.1590/0034-7167-2025-0236pt

**Authors:** Carla Maria Kortz Toledo Rodrigues, Dinair Ferreira Machado, Raissa Janine de Almeida, Hugo Fernandes, Mariana Alice Oliveira Ignacio, Ana Paula Pinho Carvalheira

**Affiliations:** I Universidade Estadual Paulista “Júlio de Mesquita Filho”. Botucatu, São Paulo, Brasil; II Universidade Federal de São Paulo. São Paulo, São Paulo, Brasil

**Keywords:** Violência contra a Mulher, Abuso de Idosos, Notificação de Abuso, Pandemia de COVID-19, Brasil.

## Abstract

**Objetivos:**

analisar os fatores associados à negligência/abandono contra mulheres idosas durante a pandemia de COVID-19 no Brasil.

**Métodos:**

estudo transversal e analítico de dados do Sistema de Informação de Agravos de Notificação de 2020-2021. Realizaram-se modelos multivariados de regressão de Poisson no *Statistical Package for the Social Sciences*.

**Resultados:**

foram notificados 5.875 casos de negligência/abandono. A maioria das vítimas era parda, viúva, sem deficiência, com baixa escolaridade e heterossexual. Os fatores associados ao aumento de negligência/abandono foram idade, possuir deficiência, autor do sexo masculino ou ambos os sexos e filho(a) ou cuidador(a) da vítima, homofobia/lesbofobia/bifobia/transfobia, conflito geracional e deficiência, e ocorrer em indústria. Por outro lado, os fatores associados à diminuição de negligência/abandono foram mulheres casadas/união consensual e uso de álcool pelo(a) autor(a).

**Conclusões:**

a notificação de violência interpessoal durante a pandemia de COVID-19 permitiu identificar vulnerabilidades e fornecer subsídios para prevenção e intervenção precoce.

## INTRODUÇÃO

Em 2020, no auge da pandemia de COVID-19, a Assembleia Geral das Nações Unidas declarou o início da Década do Envelhecimento Saudável 2020-2030. Esse período oferece uma oportunidade para reunir governos, sociedade civil, profissionais, instituições acadêmicas, meios de comunicação e o setor privado em torno de dez anos de ações em favor de uma sociedade igualitária para todas as idades, em todas as nações ao redor do mundo, com o objetivo de melhorar a vida das pessoas idosas, suas famílias e as comunidades nas quais estão inseridas^(1)^.

A população está envelhecendo em todo o mundo mais rapidamente do que no passado, e essa transição demográfica afetará quase todos os aspectos da sociedade. No mundo, já há mais de um bilhão de pessoas com 60 anos ou mais, e a maioria delas vive em países de baixa e média renda. Muitas dessas pessoas não têm sequer acesso aos recursos básicos necessários para uma vida plena e digna, enfrentando inúmeros obstáculos que as impedem de participar plenamente da sociedade^(1)^. O Brasil conta com mais de 31 milhões de pessoas idosas, o que corresponde a 16% da população. Segundo as projeções do Instituto Brasileiro de Geografia e Estatística, a porcentagem de idosos deverá dobrar nas próximas décadas^(2)^. As mulheres são maioria entre os idosos. Muitas vivem a velhice em condições de solidão e enfrentam desafios como a falta de recursos financeiros, tornando difícil atender às suas necessidades básicas, tornando-as mais suscetíveis às diversas formas de violência e aumentando a vulnerabilidade desse grupo^(3)^.

Nesse sentido, as mulheres idosas enfrentam dupla vulnerabilidade com relação à violência, decorrente tanto da idade quanto do gênero. A opressão e a dominação relacionadas à desigualdade de gênero, presentes em todas as fases da vida, intensificam-se na velhice^(4-6)^.

Segundo metanálise divulgada pela *The Lancet Global Health*, cerca de um a cada seis idosos ao redor do mundo é vítima de algum tipo de violência, correspondendo a uma prevalência de 15,7%, sendo 11,6% de violência psicológica, 6,8% de abuso financeiro, 2,6% de violência física, 0,9% de violência sexual e 4,2% de negligência/abandono^(7)^.

A violência contra a mulher idosa, manifestada principalmente por negligência e abandono, revela as falhas estruturais de uma sociedade que, historicamente, marginaliza tanto os idosos quanto as mulheres, formando um grupo altamente vulnerável e suscetível a fatores externos, como a pandemia de COVID-19, que intensificou os índices de violência contra mulheres e idosos^(8-10)^.

Com o advento da COVID-19, algumas ações tiveram de ser tomadas devido à alta taxa de contágio do vírus. Para reduzir a velocidade de transmissão, uma série de intervenções foi implementada, como distanciamento social e isolamento^(11)^. Neste contexto, evidenciou-se o *lockdown* como um potencializador da violência contra mulheres e idosos, implicando um aumento exponencial em nível mundial^(12,13)^.

Em relação à violência contra mulher, países como Argentina, Canadá, França, Alemanha, Reino Unido e Estados Unidos denunciaram o aumento dos casos e da demanda por abrigos de emergência e redes de apoio. No Reino Unido, Austrália e França, as denúncias de violência subiram 65%^(14)^. Na Índia, as denúncias dobraram na primeira semana de distanciamento^(15)^. Países da América Latina, como Bolívia, Colômbia, Argentina e Brasil, também registraram uma intensificação da violência doméstica^(16)^. No Brasil, o Ligue 180 registrou um aumento de 35,9% no número de ligações recebidas em abril de 2020, totalizando 9.842 chamadas em comparação a abril de 2019^(17)^.

Durante a pandemia, houve um aumento de dez vezes em abusos e negligências contra idosos, especialmente contra aqueles com problemas de mobilidade e dificuldades de acesso a informações^(6)^. Porém, apesar do aumento alarmante, o tema ainda é pouco estudado, com uma carência de pesquisas e discussões que abordem o assunto em específico^(4,8,18)^.

É inegável que o isolamento social, imposto pela pandemia, acentuou as fraquezas estruturais da sociedade, levando a um aumento expressivo dos casos de negligência e abandono, particularmente em mulheres idosas. Contudo, tais fenômenos ainda carecem de maior investigação científica, sendo que a literatura disponível apresenta lacunas significativas em relação à violência contra os idosos no período pandêmico, especialmente no que tange às idosas, que enfrentam desafios adicionais devido à interseção entre idade e gênero^(3,13,19)^.

Diante do exposto, surgiu nos autores o seguinte questionamento: quais são os aspectos associados à negligência/abandono contra mulheres idosas durante a pandemia de COVID-19?

## OBJETIVOS

Analisar os fatores associados à negligência/abandono contra mulheres idosas durante a pandemia de COVID-19 no Brasil.

## MÉTODOS

### Aspectos éticos

Foi assegurada a preservação dos aspectos éticos, de acordo com a Resolução do Conselho Nacional de Saúde nº 510, de 7 de abril de 2016, parágrafo único, que apresenta que não serão registradas nem avaliadas pelo sistema Comitê de Ética em Pesquisa/Comissão Nacional de Ética em Pesquisa (CEP/CONEP), no item II, pesquisa que utilize informações de acesso público, nos termos da Lei no 12.527, de 18 de novembro de 2011.

Destaca-se que o banco de dados utilizado é de acesso público, e não contém o nome das participantes ou qualquer outra possibilidade de identificação individual das mulheres, de forma a garantir o anonimato. Assim, não foi necessário encaminhamento para apreciação do CEP.

### Desenho, período e local do estudo

Trata-se de estudo transversal^(20)^ e analítico, guiado pela ferramenta *STrengthening the Reporting of OBservational studies in Epidemiology* ^(21)^, desenvolvido a partir de dados secundários provenientes do Sistema de Informação de Agravos de Notificação (SINAN), obtidos a partir do Departamento de Informática do Sistema Único de Saúde (DATASUS) e acessados por meio do TabNet no dia 4 de março de 2024.

### População, critérios de inclusão e exclusão

Foram incluídos todos os casos de negligência/abandono contra mulher idosa ocorridos no Brasil e registrados no SINAN entre 2020 e 2021. As notificações em que o gênero da vítima foi “ignorado” e de casos suspeitos ou confirmados de violência autoprovocada foram excluídas.

### Protocolo do estudo

Os dados foram coletados por meio das informações apontadas nas fichas de notificação de casos suspeitos ou confirmados de violência interpessoal do SINAN, disponibilizados pela Secretaria Nacional de Vigilância em Saúde através do TabNet, um programa desenvolvido pelo DATASUS, e estão acessíveis *online* sem restrições de acesso. Na ocasião da busca, o sistema possuía dados disponíveis até o ano de 2022.

Foram analisadas variáveis relacionadas às características sociodemográficas das mulheres idosas, do(a) autor(a) e da negligência/abandono, apresentadas no Quadro 1.

**Tabela 1:** Notificações de negligência/abandono contra mulher idosa segundo características sociodemográficas das vítimas, Brasil, 2020 - 2021

Características sociodemográficas	n	%
**Raça/cor**		
Branca	2.212	37,7
Preta	431	7,3
Amarela	43	0,7
Parda	2.867	48,8
Indígena	30	0,5
Em branco	292	5,0
**Escolaridade**		
Analfabeta	314	5,3
1ª a 4ª série incompleta do ensino fundamental	753	12,8
4ª série incompleta do ensino fundamental	286	4,9
5ª a 8ª série incompleta do ensino fundamental	306	5,2
Ensino fundamental completo	225	3,8
Ensino médio incompleto	74	1,3
Ensino médio completo	119	2,0
Educação superior incompleta	9	0,2
Educação superior completa	52	0,9
Ignorado	3.737	63,6
**Situação conjugal**		
Solteira	720	12,3
Casada/união consensual	841	14,3
Viúva	1.684	28,7
Separada	271	4,6
Em branco	2.659	40,2
Possui algum tipo de deficiência/transtorno		
Não	4.395	74,8
Sim	1.480	25,2
**Orientação sexual**		
Heterossexual	3.740	63,7
Homossexual	21	0,4
Bissexual	4	0,1
Em branco	2.110	35,9

*Fonte: Sistema de Informação de Agravos de Notificação, 2020 - 2021.*

**Quadro 1:** Variáveis do estudo segundo tipo e expressão, Brasil, 2020-2021

Variável	Tipo	Expressão
**Características sociodemográficas das mulheres idosas**		
Raça/cor	Categórica	Branca; preta; amarela; parda; indígena; em branco.
Escolaridade (anos completos)	Categórica	Analfabeta; 1ª a 4ª série incompleta do ensino fundamental; 4ª série incompleta do ensino fundamental; 5ª a 8ª série incompleta do ensino fundamental; ensino fundamental completo; ensino médio incompleto; ensino médio completo; ensino superior incompleto; ensino superior completo; ignorado.
Situação conjugal	Categórica	Solteira; casada/união consensual; viúva; separada; em branco.
Possui algum tipo de deficiência/transtorno	Dicotômica	Sim; não.
Orientação sexual	Categórica	Heterossexual; homossexual; bissexual; em branco.
**Características do(a) autor(a) e da negligência/abandono**		
Sexo do(a) autor(a)	Categórica	Feminino; masculino; ambos os sexos.
Suspeita do uso de álcool	Dicotômica	Sim; não.
Ciclo de vida do(a) autor(a)	Categórica	Criança (0-9 anos); adolescente (10-19 anos); jovem (20-24 anos); pessoa adulta (25-59 anos); pessoa idosa (≥60 anos).
Local de ocorrência	Categórica	Residência; habitação coletiva; escola; local de prática esportiva; bar ou similar; via pública; comércio/serviços; indústria/construção.
Motivação da violência	Categórica	Sexismo; homofobia/lesbofobia/bifobia/transfobia; racismo; intolerância religiosa; xenofobia; conflito geracional; situação de rua; deficiência; outros.
Relação do(a) autor(a) com a mulher idosa	Categórica	Pai; mãe; padrasto; madrasta; cônjuge; ex-cônjuge; namorado(a); ex-namorado(a); filho(a); desconhecido; irmão(a), conhecido/amigo; cuidador(a); patrão(oa)/chefe; institucional; policial/agente da lei.

### Análise dos resultados e estatística

Foram elaboradas análises descritivas das variáveis sociodemográficas das mulheres idosas, do(a) autor(a) e da negligência/abandono. A investigação dos fatores associados à negligência/abandono contra mulheres idosas no contexto pandêmico foi realizada ajustando modelos de regressão múltipla com reposta Poisson em duas etapas. Na primeira, foi ajustado um modelo de regressão bivariada, incluindo, no componente determinístico, todas as variáveis explicativas. As variáveis que apresentaram associação com p<0,05 foram consideradas na segunda etapa, que consistiu no ajuste de um novo modelo de regressão linear múltipla com resposta Poisson apenas com as variáveis identificadas na etapa anterior. No modelo final, os resultados foram expressos como razões de prevalência, com Intervalos de Confiança de 95% e valores de p, considerando p<0,05 como nível de significância estatística.

Para tabulação e análise dos dados, foram utilizados os *softwares* Microsoft Excel 2007 e *Statistical Package for the Social Sciences*, respectivamente.

## RESULTADOS

No período delineado, foram notificados 5.875 casos de mulheres idosas que sofreram negligência/abandono em todo o país. De acordo com a Tabela 1, observou-se que 48,8% (n=2.867) das mulheres se declararam pardas; 12,8% (n=753) cursaram entre a 1ª e 4ª série incompleta do ensino fundamental; 28,7% (n=1.684) eram viúvas; 78,4% (n=4.395) não possuíam deficiência/transtorno; e 63,7% (n=3.740) eram heterossexuais.

Segundo a Tabela 2, 44,1% (n=2.589) dos casos de negligência/abandono foram praticados tanto pelo sexo masculino quanto feminino. Em relação ao(à) autor(a) da violência, 62,6% (n=3.676) eram adultos (25 a 59 anos); 54,9% (n=3.225) não estavam sob suspeita do uso de álcool; 69,3% (n=4.069) tinham relação de filho(a); e 5,7% (n=337) tinham relação de cuidador(a) da vítima. Considerando o local de ocorrência, 80,5% (n=4.728) ocorreram na residência (80,5%), sendo 47,6% (n=2.798) por outros motivos, e 9,6% (n=563), por conflito geracional.

**Tabela 2:** Notificações de negligência/abandono contra a mulher idosa segundo características do(a) autor(a) e da violência, Brasil, 2020 - 2021

Características	n	%
**Sexo do(a) autor(a)**		
Masculino	1.186	20,2
Feminino	1.241	21,1
Ambos os sexos	2.589	44,1
Em branco	859	14,6
**Ciclo de vida do(a) autor(a)**		
Criança (0 a 9 anos)	27	0,5
Adolescente (10 a 19 anos)	52	0,9
Jovem (20 a 24 anos)	144	2,5
Pessoa adulta (25 a 59 anos)	3.676	62,6
Pessoa idosa (≥60 anos)	606	10,3
Em branco	1.370	23,3
**Suspeita do uso de álcool**		
Não	3.225	54,9
Sim	466	7,9
Em branco	2.184	37,2
**Relação do(a) autor(a) com a mulher idosa**		
Pai	20	0,3
Mãe	51	0,9
Padrasto	1	0,0
Madrasta	1	0,0
Cônjuge	277	4,7
Ex-cônjuge	22	0,4
Namorado(a)	4	0,1
Ex-namorado(a)	10	0,2
Filho(a)	4.069	69,3
Irmão(a)	483	4,8
Conhecido(a)	126	2,1
Desconhecido(a)	29	0,5
Cuidador(a)	337	5,7
Patrão(oa)	6	0,1
Institucional	77	1,3
Policial/agente da lei	1	0,00
**Local de ocorrência da negligência/abandono**		
Residência	4.728	80,5
Habitação coletiva	102	1,7
Escola	0	0,0
Local de prática esportiva	2	0,0
Bar ou similar	1	0,0
Via pública	135	2,3
Comércio/serviços	150	2,6
Indústrias/construção	1	0,0
Em branco	756	12,9
**Motivação da negligência/abandono**		
Sexismo	69	1,2
Homofobia/lesbofobia/bifobia/transfobia	21	0,4
Racismo	2	0,0
Intolerância religiosa	3	0,1
Xenofobia	4	0,1
Conflito geracional	563	9,6
Situação de rua	11	0,2
Deficiência	89	1,5
Outros	2.798	47,6
Em branco	2.315	39,4

*Fonte: Sistema de Informação de Agravos de Notificação, 2020 - 2021.*

**Tabela 3:** Associações bivariadas por regressão simples de Poisson para explicar a negligência/abandono contra mulher idosa na pandemia, Brasil, 2020-2021

Variáveis	RP	IC	Valor de *p*
**Idade**	1,05	1,05-1,06	**0,000**
**Raça/cor**			
Indígena	0,80	0,56-1,15	0,230
Parda	1,53	1,45-1,62	**0,000**
Amarela	1,20	0,89-1,62	0,241
Preta	1,02	0,92-1,13	0,742
Branca	1		
**Escolaridade**			
1ª a 4ª série incompleta do ensino fundamental	0,91	0,79-1,03	**0,000**
4ª série completa do ensino fundamental	0,79	0,68-0,93	**0,000**
5ª a 8ª série incompleta do ensino fundamental	0,64	0,55-0,75	**0,000**
Ensino fundamental completo	0,62	0,55-0,73	**0,000**
Ensino médio incompleto	0,42	0,33-0,54	**0,000**
Ensino médio completo	0,29	0,23-0,35	**0,000**
Educação superior incompleta	0,20	0,10-0,39	**0,004**
Educação superior completa	0,36	0,27-0,48	0,143
Analfabeta	1		
**Situação conjugal**			
Separada	0,57	0,50-0,66	**0,000**
Viúva	1,33	1,22-1,45	**0,000**
Casada/união consensual	0,56	0,51-0,62	**0,000**
Solteira	1		
**Possui alguma deficiência/transtorno**			
Sim	1,90	1,79-2,02	**0,000**
Não	1		
**Orientação sexual**			
Bissexual	0,80	0,30-2,13	0,655
Homossexual	0,54	0,35-0,82	**0,004**
Heterossexual	1		
**Sexo do(a) autor(a)**			
Ambos os sexos	7,37	6,88-7,90	**0,000**
Masculino	2,98	2,75-3,23	**0,000**
Feminino	1		
**Ciclo de vida do(a) autor(a)**			
Pessoa idosa (≥60 anos)	0,51	0,34-0,74	**0,001**
Pessoa adulta (25 a 59 anos)	0,91	0,62-1,33	0,632
Jovem (20 a 24 anos)	0,38	0,25-0,57	**0,000**
Adolescente (10 a 19 anos)	0,39	0,24-0,62	**0,000**
Criança (0 a 9 anos)	1		
**Uso de álcool**			
Sim	0,23	0,21-0,25	**0,000**
Não	1		
**Relação do(a) autor(a) com a mulher idosa**			
Pai	0,88	0,57-1,36	0,557
Mãe	1,11	0,84-1,46	0,454
Padrasto	0,26	0,04-1,87	0,182
Madrasta	0,26	0,04-1,87	0,182
Irmão(a)	1,27	1,12-1,43	**0,000**
Cônjuge	0,26	0,23-0,29	**0,000**
Ex-cônjuge	0,10	0,06-0,15	**0,000**
Namorado(a)	0,09	0,03-0,24	**0,000**
Ex-namorado(a)	0,32	0,17-0,60	**0,000**
Filho(a)	3,94	3,70-4,19	**0,000**
Amigo(a)/conhecido(a)	0,31	0,26-0,37	**0,000**
Desconhecido(a)	0,09	0,07-0,14	**0,000**
Cuidador(a)	2,64	2,37-2,95	**0,000**
Patrão(oa)/chefe	0,73	0,33-1,63	0,446
Institucional	2,07	1,66-2,60	**0,000**
Policial/agente da lei	0,11	0,02-0,81	**0,000**
**Motivação da negligência/abandono**			
Outros	17,71	13,95-22,49	**0,000**
Deficiência	14,88	10,87-20,38	**0,000**
Situação de rua	3,02	1,60-5,71	**0,001**
Conflito geracional	6,47	5,04-8,31	**0,000**
Xenofobia	12,26	4,47-33,60	**0,000**
Intolerância religiosa	6,90	2,17-21,91	**0,001**
Racismo	4,60	1,13-18,75	**0,033**
Homofobia/lesbofobia/bifobia/transfobia	14,85	9,11-24,21	**0,000**
Sexismo	1		
**Local de ocorrência da negligência/abandono**			
Indústrias/construção	1,70	0,24-12,07	0,596
Comércio/serviços	1,81	1,54-2,13	**0,000**
Via pública	0,51	0,43-0,60	**0,000**
Bar ou similar	0,05	0,01-0,33	**0,002**
Local de prática esportiva	0,38	0,09-1,51	0,169
Escola	#		
Habitação coletiva	1,95	1,60-2,37	**0,000**
Residência	1		

*RP - Regressão de Poisson; IC - Intervalo de Confiança; #Estimativa não obtida por problemas de convergência do estimador de máxima verossimilhança, ou seja, estimativa não obtida por razões de cálculo.*

*Fonte: Sistema de Informação de Agravos de Notificação, 2020 - 2021.*

Na análise de regressão bivariada (Tabela 3), de maneira independente, houve associação significativa da negligência/abandono contra a mulher idosa durante a pandemia, aumentando sua ocorrência com as seguintes variáveis: ser idosa; ser parda; ser viúva; possuir algum tipo de deficiência/transtorno; o autor da violência ser do sexo masculino e ambos os sexos; a relação entre o(a) autor(a) e a vítima ser filho(a), irmão(a), cuidador(a) e institucional; a motivação que levou à negligência/abandono ser por outros, deficiências, situação de rua, conflito geracional, xenofobia, intolerância religiosa, racismo e homofobia/lesbofobia/bifobia/transfobia; e em relação ao local da ocorrência ser em comércio/serviços e habitação coletiva.

**Tabela 4:** Análise de regressão múltipla de Poisson para explicar a negligência/abandono contra mulher idosa na pandemia, Brasil, 2020-2021

Variáveis	RP	IC	Valor de *p*
**Idade**	1,03	1,02-1,04	**0,000**
**Raça/cor**			
Indígena	0,85	0,33-2,12	0,728
Parda	1,15	0,95-1,40	0,137
Amarela	0,56	0,79-4,08	0,574
Preta	1,05	0,78-1,43	0,715
Branca	1		
**Escolaridade**			
1ª a 4ª série incompleta do ensino fundamental	0,80	0,45-1,45	0,480
4ª série completa do ensino fundamental	0,54	0,13-2,25	0,404
5ª a 8ª série incompleta do ensino fundamental	0,77	0,51-1,15	0,212
Ensino fundamental completo	0,72	0,42-1,24	0,244
Ensino médio incompleto	0,97	0,66-1,42	0,881
Ensino médio completo	1,02	0,72-1,43	0,902
Educação superior incompleta	1,01	0,73-1,40	0,911
Educação superior completa	1,06	0,81-1,38	0,658
Analfabeta	1		
**Situação conjugal**			
Separada	0,71	0,50-1,01	0,060
Viúva	0,78	0,61-1,00	0,054
Casada/união consensual	0,69	0,52-0,93	**0,015**
Solteira	1		
**Possui alguma deficiência/transtorno**			
Sim	1,75	1,31-2,33	**0,000**
Não	1		
**Orientação sexual**			
Bissexual	0,53	0,07-3,88	0,535
Homossexual	1,13	0,41-3,06	0,808
Heterossexual	1		
**Sexo do(a) autor(a)**			
Ambos os sexos	2,02	1,52-2,67	**0,000**
Masculino	1,51	1,20-1,89	**0,000**
Feminino	1		
**Ciclo de vida do(a) autor(a)**			
Pessoa idosa	1,46	0,20-10,69	0,708
Pessoa adulta	1,48	0,20-10,70	0,697
Jovem	0,89	0,11-6,85	0,918
Adolescente	0,94	0,10-8,31	0,961
Criança	1		
**Uso de álcool**			
Sim	0,39	0,30-0,50	**0,000**
Não	1		
**Relação do(a) autor(a) com a mulher idosa**			
Irmão(a)	1,28	0,83-1,99	0,255
Cônjuge	1,07	0,70-1,63	0,740
Ex-cônjuge	0,60	0,21-1,67	0,334
Namorado(a)	#		
Ex-namorado(a)	1,69	0,23-12,38	0,601
Filho(a)	2,52	1,92-3,30	**0,000**
Amigo(a)/conhecido(a)	0,66	0,40-1,08	0,099
Desconhecido(a)	0,45	0,16-1,25	0,126
Cuidador(a)	1,52	1,05-2,19	**0,025**
Patrão(oa)/chefe	#		
Institucional	1,63	0,34-7,78	0,539
Policial/agente da lei	#		
**Motivação da negligência/abandono**			
Outros	5,51	3,24-9,36	**0,000**
Deficiência	4,98	2,45-10,10	**0,000**
Situação de rua	1,38	0,17-11,02	0,756
Conflito geracional	3,78	2,20-6,50	**0,000**
Xenofobia	#		
Intolerância religiosa	4,30	0,56-33,98	0,160
Racismo	5,45	0,70-46,45	0,105
Homofobia/lesbofobia/bifobia/transfobia	5,81	1,65-20,46	**0,006**
Sexismo	1		
**Local de ocorrência da negligência/abandono**			
Indústrias/construção	8,68	1,18-63,87	**0,034**
Comércio/serviços	1,35	0,72-2,53	0,335
Via pública	1,11	0,65-1,91	0,686
Bar ou similar	#		
Local de prática esportiva	#		
Escola	#		
Habitação coletiva	1,17	0,43-3,18	0,746
Residência	1		

*RP - Regressão de Poisson; IC - Intervalo de Confiança; #Estimativa não obtida por problemas de convergência do estimador de máxima verossimilhança, ou seja, estimativa não obtida por razões de cálculo.*

*Fonte: Sistema de Informação de Agravos de Notificação, 2020 - 2021.*

Por outro lado, também de maneira independente, houve associação significativa da negligência/abandono contra a mulher idosa durante a pandemia, diminuindo sua ocorrência, com as seguintes variáveis: ser mulheres com escolaridade da 1ª a 4ª série ao ensino superior incompleto; ser idosa separada e casada/união consensual; ser homossexual; em relação ao ciclo de vida do(a) autor(a), ser idoso (≥60 anos), jovem (20 a 24 anos) e adolescente (10 a 19 anos); estar alcoolizado no momento da ocorrência; a relação entre o(a) autor(a) e a vítima ser de cônjuge, ex-cônjuge, namorado(a), ex-namorado(a), policial/agente da lei e amigo/conhecido; e ter ocorrido em via pública e bar ou similar.

Na análise de regressão múltipla (Tabela 4), de maneira independente, mantiveram associação significativa com a negligência/abandono contra a mulher idosa na pandemia, aumentando sua ocorrência as seguintes variáveis: ser idosa; possuir algum tipo de deficiência/transtorno; o autor da violência ser do sexo masculino e ambos os sexos; a relação entre o(a) autor(a) e a vítima ser de filho(a) e cuidador(a); os motivos da negligência/abandono serem homofobia/lesbofobia/bifobia/transfobia, conflito geracional, deficiências e outros; e a violência ocorrer em indústria. Por outro lado, também de maneira independente, mantiveram associação significativa com a negligência/abandono contra a mulher idosa na pandemia, diminuindo sua ocorrência, as seguintes variáveis: mulheres casadas/união consensual; e o(a) autor(a) estar em suspeita de uso de álcool.

## DISCUSSÃO

Evidências apontam que idosas com dependência para atividades diárias ou com demência estão mais suscetíveis a abuso e negligência, já que a sobrecarga dos cuidadores, muitas vezes sem preparo adequado, pode levar à negligência ou abandono, intencional ou não. Idosos com transtornos neuro cognitivos, em particular, são uma população de alto risco para abuso no contexto de cuidado. Essas condições de saúde crônicas, juntamente com limitações físicas e cognitivas, aumentam a vulnerabilidade dessas mulheres à violência^(8,9,22-24)^.

Durante a pandemia, a ocorrência de violência cometida por agressores do sexo masculino aumentou, como observado nesta pesquisa. Estudo em São Paulo indicou que 56,7% dos casos de violência contra idosos no primeiro ano da pandemia tiveram agressores do sexo masculino, com a frustração devido à perda de emprego ou dificuldades econômicas sendo frequentemente direcionada às idosas^(9)^. No entanto, mulheres também podem ser perpetradoras de violência, especialmente quando enfrentam sobrecarga emocional e econômica no cuidado das idosas. Nesse contexto, cuidadoras sem apoio adequado frequentemente negligenciam suas responsabilidades, resultando em abandono involuntário^(25)^.

Contudo, em sua maioria, os(as) cuidadores(as) são familiares mulheres, em geral filhas ou noras com vínculo afetivo e estreito com a idosa. O trabalho de cuidado, muitas vezes, é contínuo e solitário, sem o suporte de serviços ou políticas públicas de assistência. Essa função impõe limitações à vida pessoal do(a) cuidador(a), gerando sobrecarga, desemprego, e distanciamento da rede social e afetiva gerando relações conflituosas dentro da família e com a idosa. A desigualdade de gênero nitidamente atravessa essas relações, pois, em algum momento, essa idosa é quem foi a responsável pelo cuidado familiar, e ao transitar para condição de dependente dos cuidados, torna-se inútil ao sistema e um peso à família^(26)^.

Esse padrão reflete a feminização da velhice, na qual as mulheres idosas vivem mais, mas enfrentam maior dependência funcional e, consequentemente, maior ocorrência de abandono e negligência. Assim, o contexto pandêmico mostrou que ambos os sexos podem atuar como agressores, embora as dinâmicas e as motivações possam variar^(24)^.

Outro aspecto relacionado a violência são os preconceitos relacionados à orientação sexual e identidade de gênero. A violência contra mulheres idosas Lésbicas, Gays, Bissexuais, Transexuais/Travestis, *Queer*, Intersexo, Assexual e demais orientações sexuais e identidades de gênero é intensificada por preconceitos como homofobia e transfobia, que afetam seu acesso a cuidados, especialmente em ambientes familiares e de saúde. Durante a pandemia, essas discriminações aumentaram, resultando em maior rejeição e abandono familiar, além do isolamento social, que agravou a exclusão de serviços de apoio. O envelhecimento dessas mulheres, somado aos estigmas relacionados à idade e à falta de aceitação familiar e social, torna-as mais vulneráveis à negligência e abandono. Na pandemia, a invisibilidade social e a falta de políticas de proteção específicas amplificaram ainda mais essa situação^(13,27)^.

Considerando o local de ocorrência da negligência/abandono na pesquisa atual, e embora não haja estudos recentes que identifiquem diretamente as indústrias como fatores que aumentam a prevalência de negligência/abandono em mulheres idosas, a violência no ambiente de trabalho, incluindo abandono gerencial, afeta a permanência de idosos no mercado. A falta de preparo das empresas para lidar com o envelhecimento da força de trabalho e os estereótipos negativos resultam em discriminação e abandono precoce, configurando violência estrutural. Durante a pandemia de COVID-19, esses fatores pioraram, especialmente para mulheres idosas, que enfrentaram maior vulnerabilidade ao abandono no trabalho e na família devido ao isolamento social e dificuldades econômicas^(28)^.

Ao longo da pandemia, a ocorrência de negligência/abandono foi menor nas mulheres idosas casadas ou em união estável, pois a presença de um cônjuge muitas vezes fornece suporte emocional e prático. O estado civil pode ser um fator para redução da ocorrência de negligência, pois os casais tendem a desenvolver relações de cuidado mútuo, o que pode atenuar a ocorrência de abandono. Idosas que viviam com seus cônjuges relataram menor isolamento emocional, pois o parceiro estava presente para oferecer apoio imediato. Além disso, a estabilidade financeira de muitos casais contribuiu para manter o acesso a serviços essenciais durante a pandemia, reduzindo a ocorrência de negligência^(8)^.

A variável suspeita de uso de álcool pelo(a) autor(a) da violência também se associou à menor prevalência de negligência/abandono. Isso sugere que a estrutura familiar e contextos que envolvem o consumo de álcool podem influenciar a dinâmica da negligência/abandono. Uma possível explicação é que, durante a pandemia, houve restrição do funcionamento de bares e outros estabelecimentos públicos. Dessa forma, o consumo de álcool, que era realizado em espaços como esses, passou ao privado, tendo a residência se tornado o local de escolha para essa prática^(29)^.

### Limitações do estudo

A principal limitação do estudo inclui a possibilidade de incremento de notificações tardias de casos de violência interpessoal no SINAN, podendo não compor as informações coletadas neste estudo. Além disso, foi alto o número de notificações em que as variáveis foram marcadas como “ignorado” ou “em branco”. Esse tipo de ocorrência é considerado uma falha no preenchimento da ficha de notificação compulsória de casos suspeitos ou confirmados de violência, pois afeta significativamente a caracterização precisa do perfil das vítimas e a adoção adequada de medidas de proteção e enfrentamento^(30)^.

Outro achado relevante foi que nem todas as informações presentes na ficha de notificação compulsória estavam tabuladas pelo sistema, tais como os desfechos ou encaminhamentos dos casos. No entanto, essa fragilidade não compromete os resultados, pois são apresentadas informações relevantes sobre as características da negligência e abandono sofridos por mulheres idosas durante a pandemia de COVID-19 no Brasil. Por fim, a delimitação da coleta de dados aos anos de 2020 e 2021, por representarem o período pandêmico, impossibilita a análise de mudanças no pós-pandemia, evidenciando a relevância de novas investigações.

### Contribuições para a área da enfermagem, saúde ou políticas públicas

Destaca-se a importância da notificação compulsória de violência interpessoal durante a pandemia de COVID-19, pois ela possi----bilitou identificar as mulheres idosas com maior vulnerabilidade, promovendo subsídios para prevenção e intervenção precoce nos casos de violência contra a mulher idosa.

Tendo em vista o elevado número de variáveis com dados “em branco” e “ignorado” encontrados, sugere-se a necessidade de educação permanente em todo território nacional sobre o preenchimento correto da ficha de notificação de casos suspeitos ou confirmados de violência.

## CONCLUSÕES

O presente estudo viabilizou a análise dos fatores associados à negligência/abandono de mulheres idosas durante a pandemia de COVID-19 no Brasil. O aumento de sua ocorrência esteve associado significativamente à idade da mulher, presença de deficiência/transtorno, perfil do agressor (sexo masculino ou ambos os sexos) e relação com a vítima, especialmente quando se tratava de filho(a) ou cuidador(a). Motivações como homofobia/lesbofobia/bifobia/transfobia, conflitos geracionais e deficiência também se associaram, assim como a ocorrência em ambientes industriais. Por outro lado, ser casada/união consensual e a suspeita de uso de álcool pelo agressor diminuíram, de maneira independente, a ocorrência desse agravo.

Esses achados reforçam a necessidade de estratégias intersetoriais que considerem as especificidades desse perfil, promovendo políticas públicas voltadas à proteção e ao cuidado das mulheres idosas em contextos de vulnerabilidade.

## Data Availability

Os dados de pesquisa só estão disponíveis mediante solicitação.
